# Accuracy of Magnetic Resonance Imaging–Guided Biopsy to Verify Breast Cancer Pathologic Complete Response After Neoadjuvant Chemotherapy: A Nonrandomized Controlled Trial

**DOI:** 10.1001/jamanetworkopen.2020.34045

**Published:** 2021-01-15

**Authors:** Elizabeth J. Sutton, Lior Z. Braunstein, Mahmoud B. El-Tamer, Edi Brogi, Mary Hughes, Yolanda Bryce, Jill S. Gluskin, Simon Powell, Alyssa Woosley, Audree Tadros, Varadan Sevilimedu, Danny F. Martinez, Larowin Toni, Olga Smelianskaia, C. Gregory Nyman, Pedram Razavi, Larry Norton, Maggie M. Fung, James D. Sedorovich, Virgilio Sacchini, Elizabeth A. Morris

**Affiliations:** 1 Department of Radiology, Memorial Sloan Kettering Cancer Center, New York, New York; 2 Department of Radiation Oncology, Memorial Sloan Kettering Cancer Center, New York, New York; 3 Department of Surgery, Memorial Sloan Kettering Cancer Center, New York, New York; 4 Department of Pathology, Memorial Sloan Kettering Cancer Center, New York, New York; 5 Department of Epidemiology and Biostatistics, Memorial Sloan Kettering Cancer Center, New York, New York; 6 Department of Medicine, Memorial Sloan Kettering Cancer Center, New York, New York; 7 GE Healthcare, New York, New York

## Abstract

**Importance:**

After neoadjuvant chemotherapy (NAC), pathologic complete response (pCR) is an optimal outcome and a surrogate end point for improved disease-free and overall survival. To date, surgical resection remains the only reliable method for diagnosing pCR.

**Objective:**

To evaluate the accuracy of magnetic resonance imaging (MRI)–guided biopsy for diagnosing a pCR after NAC compared with reference-standard surgical resection.

**Design, Setting, and Participants:**

Single-arm, phase 1, nonrandomized controlled trial in a single tertiary care cancer center from September 26, 2017, to July 29, 2019. The median follow-up was 1.26 years (interquartile range, 0.85-1.59 years). Data analysis was performed in November 2019. Eligible patients had (1) stage IA to IIIC biopsy-proven operable invasive breast cancer; (2) standard-of-care NAC; (3) MRI before and after NAC, with imaging complete response defined as no residual enhancement on post-NAC MRI; and (4) definitive surgery. Patients were excluded if they were younger than 18 years, had a medical reason precluding study participation, or had a prior history of breast cancer.

**Interventions:**

Post-NAC MRI-guided biopsy without the use of intravenous contrast of the tumor bed before definitive surgery.

**Main Outcomes and Measures:**

The primary end point was the negative predictive value of MRI-guided biopsy, with true-negative defined as negative results of the biopsy (ie, no residual cancer) corresponding to a surgical pCR. Accuracy, sensitivity, positive predictive value, and specificity were also calculated. Two clinical definitions of pCR were independently evaluated: definition 1 was no residual invasive cancer; definition 2, no residual invasive or in situ cancer.

**Results:**

Twenty of 23 patients (87%) had evaluable data (median [interquartile range] age, 51.5 [39.0-57.5] years; 20 women [100%]; 13 White patients [65%]). Of the 20 patients, pre-NAC median tumor size on MRI was 3.0 cm (interquartile range, 2.0-5.0 cm). Nineteen of 20 patients (95%) had invasive ductal carcinoma; 15 of 20 (75%) had stage II cancer; 11 of 20 (55%) had *ERBB2* (formerly *HER2* or *HER2/*neu)–positive cancer; and 6 of 20 (30%) had triple-negative cancer. Surgical pathology demonstrated a pCR in 13 of 20 (65%) patients and no pCR in 7 of 20 patients (35%) when pCR definition 1 was used. Results of MRI-guided biopsy had a negative predictive value of 92.8% (95% CI, 66.2%-99.8%), with accuracy of 95% (95% CI, 75.1%-99.9%), sensitivity of 85.8% (95% CI, 42.0%-99.6%), positive predictive value of 100%, and specificity of 100% for pCR definition 1. Only 1 patient had a false-negative MRI-guided biopsy result (surgical pathology showed <0.02 cm of residual invasive cancer).

**Conclusions and Relevance:**

This study’s results suggest that the accuracy of MRI-guided biopsy to diagnose a post-NAC pCR approaches that of reference-standard surgical resection. MRI-guided biopsy may be a viable alternative to surgical resection for this population after NAC, which supports the need for further investigation.

**Trial Registration:**

ClinicalTrials.gov Identifier: NCT03289195

## Key Points

**Question:**

Is the accuracy of magnetic resonance imaging (MRI)–guided biopsy comparable with reference-standard surgical resection for diagnosing pathologic complete response after neoadjuvant chemotherapy in patients with breast cancer?

**Findings:**

In this pilot nonrandomized controlled trial of 20 patients with evaluable data, the accuracy of MRI-guided biopsy for diagnosing pathologic complete response after neoadjuvant chemotherapy was 95% and the negative predictive value was 92.8%.

**Meaning:**

The results from this pilot study suggest greater accuracy with this method than do the majority of published data and support the need for a larger study comparing MRI-guided biopsy with reference-standard surgical resection in diagnosing a pathologic complete response after neoadjuvant chemotherapy.

## Teaser

This nonrandomized controlled trial uses data from a single tertiary care center in the US to investigate the accuracy of magnetic resonance imaging (MRI)–guided biopsy compared with surgical resection for assessing pathologic complete response after neoadjuvant chemotherapy in patients with breast cancer.

## Introduction

Breast cancer is the most common noncutaneous malignant neoplasm,^1^ the management of which has traditionally used a triple-modality approach in the form of surgery after adjuvant chemotherapy and radiotherapy. Randomized clinical evidence of therapeutic equivalence between neoadjuvant chemotherapy (NAC) and adjuvant chemotherapy led to significant changes in clinical practice.^2,3,4^ Neoadjuvant chemotherapy (ie, systemic therapy administered before surgery) allows for real-time response assessment and can downstage breast cancer, reducing the extent of local excision.^5^

The clinical implementation of NAC has facilitated breast-conserving surgery (lumpectomy) and sentinel lymph node biopsy for women who historically would require mastectomy and axillary lymph node dissection.^2,3^ The optimal outcome after NAC is a pathologic complete response (pCR), which is defined as no remaining invasive or in situ cancer in the breast. A pCR is a surrogate end point that is a marker for improved disease-free and overall survival.^4,6^ The likelihood of achieving pCR is largely based on breast cancer subtype (clinically defined by immunohistochemical surrogates of estrogen receptor, progesterone receptor, and *ERBB2* (formerly *HER2* or *HER2*/neu) amplification, with *ERBB2*-positive and triple-negative subtypes having the highest likelihood of pCR of up to 66.2%^7^ and 37%,^8^ respectively.

The National Comprehensive Cancer Network and American Society of Clinical Oncology guidelines currently recommend breast magnetic resonance imaging (MRI) before and after NAC as the most accurate imaging modality for monitoring treatment response and identifying pCR after NAC, with a reported accuracy of 83%.^2,9,10,11,12^ Although more accurate than mammography, ultrasonography, and clinical breast examination, MRI is not yet sufficient to obviate the need for pathologic confirmation of pCR. Thus, surgical resection is required even for the subset of patients in whom no viable tumor is ultimately detected in the surgical specimen.

Whereas the traditional approach to identifying a pCR has required definitive breast surgery after NAC (eg, lumpectomy or mastectomy), there are limited options for the noninvasive determination of residual in-breast disease. Although several clinical trials have reported the potential utility of image-guided biopsies as an alternative to surgery to diagnose pCR after NAC,^13,14,15^ all except the trial by Rauch et al^14^ demonstrated a negative predictive value (NPV) of biopsy to diagnose pCR of 76.7% or less, which is not sufficient to obviate surgery. Notably, these trials did not investigate the utility of MRI or MRI-guided biopsy. In this pilot study, we sought to evaluate the accuracy of MRI-guided biopsy for diagnosing a post-NAC pCR compared with reference-standard breast surgery.

## Methods

### Patients

**Figure 1.**
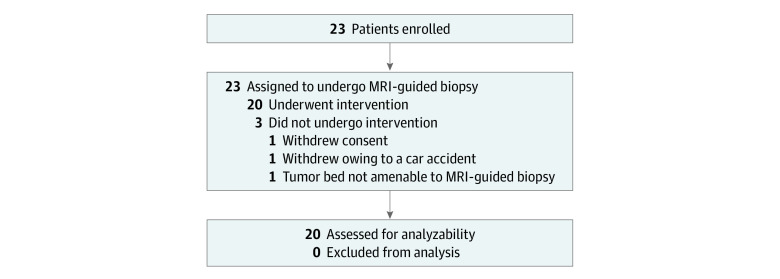
Diagram of Participant Flow Through Trial MRI indicates magnetic resonance imaging.

**Figure 2.**
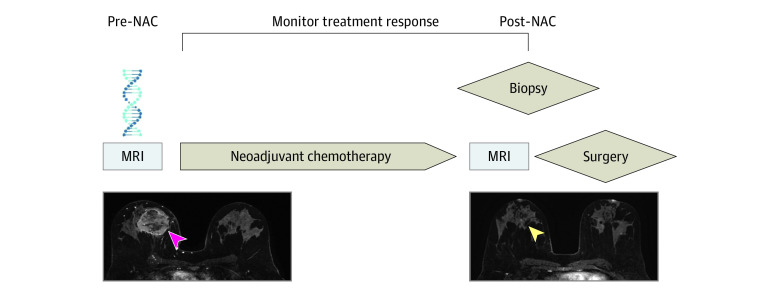
Schematic of This Single-Arm, Phase 1, Single-Institution Clinical Trial Biopsy-proven cancer of the right breast is shown in the pre–neoadjuvant chemotherapy (pre-NAC) image (magenta arrowhead); complete response is shown in the post-NAC image (yellow arrowhead). MRI indicates magnetic resonance imaging.

This was a single-arm, phase 1, nonrandomized, single-institution study performed at a tertiary cancer center in the US that was approved by the Memorial Sloan Kettering Cancer Center institutional review board and followed the Transparent Reporting of Evaluations With Nonrandomized Designs (TREND) reporting guidelines.^16^ Written informed consent was obtained for each patient. The full trial protocol is included in Supplement 1. A total of 23 patients were enrolled from September 26, 2017, to July 29, 2019, and 20 of 23 patients (87%) had evaluable data (Figure 1). We included patients with (1) American Joint Committee on Cancer stages IA to IIIC; (2) biopsy-proven, operable invasive breast cancer; (3) tumor bed suitable for post-NAC MRI-guided biopsy as determined by a breast radiologist; (4) completed standard-of-care NAC; (5) completed pre- and post-NAC MRIs; (6) imaging complete response on post-NAC MRI defined as no residual contrast enhancement; and (7) completed definitive surgery at our institution. Exclusion criteria included age less than 18 years, medical reason precluding study participation, and prior history of breast cancer. Figure 2 is a schematic of this clinical trial.

### MRI-Guided Biopsy

**Figure 3.**
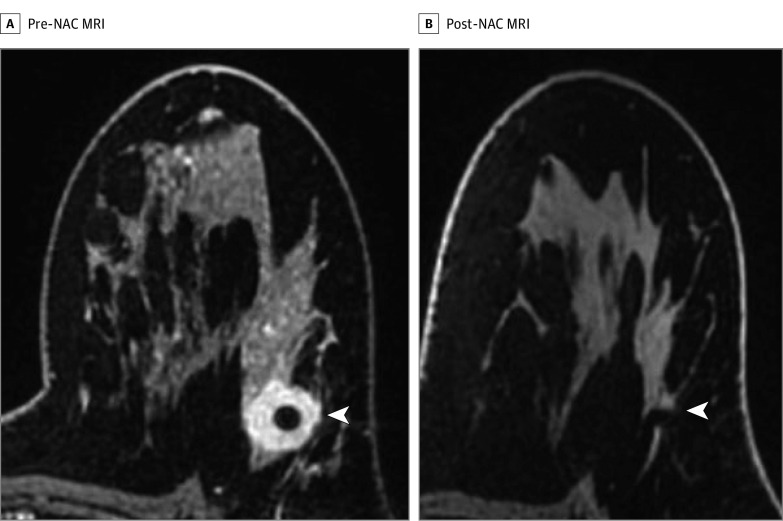
Magnetic Resonance Imaging (MRI) Before and After Neoadjuvant Chemotherapy (NAC) Demonstrating an Imaging Complete Response After NAC A, Axial fat-saturated T1-weighted postcontrast imaging of a biopsy-proven left breast *ERBB2* (formerly *HER2* or *HER2*/neu)–positive invasive ductal carcinoma (arrowhead). B, Axial fat-saturated T1-weighted postcontrast imaging of the same patient demonstrating an imaging complete response defined as no residual tumor enhancement in the treated tumor bed as indicated by the accurately positioned biopsy marker (arrowhead) and anatomic landmarks.

All MRI-guided biopsies were performed with a 1.5- or 3.0-T whole-body MRI unit (GE Discovery 450w or 750; GE Medical Systems) equipped with a dedicated 8- or 16-channel surface Sentinelle breast coil (Sentinelle Vanguard). Standard-of-care MRI-guided biopsy was performed as previously described^17^ except that no intravenous gadolinium was used for this study. Biopsy target was the treated tumor bed defined by the accurately positioned pre-NAC marker at the site of biopsy-proven cancer, anatomic landmarks, or both. Figure 3 is a representative case showing an imaging complete response after NAC. A 9-gauge vacuum-assisted, MRI-compatible biopsy system (ATEC Breast Biopsy System; Suros Surgical Systems) was used. Through a single incision site, 7 to 12 samples were taken and sent to pathology for analysis. A postbiopsy titanium marker was placed under MRI guidance, and postbiopsy mammography was performed to document representative sampling of the treated tumor bed and adequate positioning of the biopsy marker. The study intervention was defined by the predetermined, nonstudy surgical date. MRI-guided biopsy was performed 0 to 60 days after completing NAC and before surgery.

### Follow-up and End Points

Results of the MRI-guided biopsy and reference-standard surgical pathological examination were interpreted by breast pathologists, and the Miller-Payne grading system was used to assess tumor response including pCR. Our primary end point was the NPV of MRI-guided biopsy, with a true-negative finding defined as a negative biopsy result (no residual cancer) that corresponded to a surgical pCR. Accuracy, sensitivity, positive predictive value (PPV), and specificity were also calculated. Two clinical definitions of pCR were independently evaluated: definition 1 was no residual invasive cancer and definition 2 was no residual invasive or in situ cancer. Other covariates included patient age and breast cancer subtype defined by immunohistochemical surrogates (ie, estrogen hormone receptor, progesterone hormone receptor, and *ERBB2* positive or negative). Accuracy was defined as the ratio of the total number of correct test results (both true-positive and true-negative) to the sample size. Protocol-specified adverse events were defined, and no protocol-specified adverse events were reported.

### Statistical Analysis

For this study, we considered an acceptable NPV to be 85%. With a 1-sided type I error rate of .05 and a statistical power of 95%, a sample of 20 patients was required to detect an acceptable NPV of 85%. Sample size calculation was done using the clinfun package in R, version 3.5.2 (R Foundation) with the ph2single function. We anticipated that 14 of 20 patients (70%) would have a negative (pCR) MRI-guided biopsy result. We estimated NPV, sensitivity, specificity, PPV, and accuracy using PROC SURVEYFREQ in SAS, version 9.4 (SAS Institute Inc). As an exploratory analysis, we performed a logistic regression analysis and receiver operating characteristic (ROC) curve analysis using the PROC LOGISITIC statement in SAS, version 9.4, with Firth correction. The Firth correction is used to adjust for quasi-complete separation of data points—a statistical phenomenon that occurs when certain values of risk factors or combination of risk factors are almost only associated with a single outcome category, thereby allowing for easy separability of these factors by the outcome variable.^18^ Logistic regression analysis was performed using surgical pathology findings as the outcome variable and MRI-guided biopsy pathology results, age, and breast cancer subtype as explanatory variables of interest. A lower limit for the 95% CI of the area of the ROC curve of greater than 0.5 was used to suggest that the MRI-guided biopsy is diagnostically accurate. We chose a lower threshold for the lower bound of the 95% CI for the area under the ROC curve, anticipating a wider CI, which can be mainly attributed to the small sample size. Kendall ranked correlation coefficient was calculated to test for the correlation between MRI-guided biopsy result and reference-standard surgical resection findings. The level of statistical significance was set at an α of .05.

## Results

### Patient Characteristics

Of the 23 patients who were enrolled from September 26, 2017, to July 29, 2019, into the trial, 20 (87%) had evaluable data (median [interquartile range] age, 51.5 [39.0-57.5] years; 20 women [100%]; 13 White patients [65%]). Of the 20 patients, the pre-NAC median tumor size on MRI was 3.0 cm (interquartile range, 2.0-5.0 cm); 19 patients (95%) had invasive ductal carcinoma, 15 (75%) had stage II cancer, 11 (55%) had *ERBB2*-positive cancer, and 6 (30%) had triple-negative cancer. Surgical pathology findings demonstrated a pCR in 13 of 20 patients (65%) and no pCR in 7 (35%) when pCR definition 1 was used. The median follow-up for patients with evaluable data was 1.26 years (interquartile range, 0.85-1.59 years). Patient characteristics are included in Table 1.

**Table 1.** Characteristics of 20 Patients With Evaluable Data Who Underwent Post-NAC MRI-Guided Biopsy

Characteristic	Value, No. (%)^a^
Age, median (IQR), y	51.5 (39.0-57.5)
Race/ethnicity	
Asian	3 (15)
Black or African American	4 (20)
White	13 (65)
Sex	
Men	0
Women	20 (100)
Stage	
IA	1 (5)
IB	0
IIA	7 (35)
IIB	8 (40)
IIIA	2 (10)
IIIB	0
IIIC	2 (10)
IV	0
Tumor size before NAC, median (IQR), cm	3.0 (2.0-5.0)
Invasive histologic type	
Ductal	19 (95)
Lobular	1 (5)
HR and *ERBB2* status	
HR+ *ERBB2*−	3 (15)
HR+ *ERBB2*+	7 (35)
HR− *ERBB2*+	4 (20)
HR− *ERBB2*−	6 (30)
Surgery	
Lumpectomy	12 (60)
Mastectomy	8 (40)
Nodal status	
Positive	6 (30)
Negative	14 (70)
Follow-up duration, median (IQR), y	1.26 (0.85-1.59)
Recurrence	
Yes	1 (5)
No	19 (95)
Survival	
Dead of disease	1 (5)
Alive, no evidence of disease	19 (95)

Abbreviations: HR, hormone receptor; IQR, interquartile range; MRI, magnetic resonance imaging; NAC, neoadjuvant chemotherapy; −, negative; +, positive.

^a^ Values are listed as number (percentage) unless specified otherwise.

### Pathologic Complete Response

Breast cancer NAC response and the accuracy of MRI-guided biopsy for diagnosing a pCR are given in Table 2. Of the 20 patients with evaluable data, surgical pathology results demonstrated residual invasive cancer in 8 patients (40%), ductal carcinoma in situ but no invasive cancer in 1 patient (5%), and no residual invasive or in situ cancer in 11 patient (55%). The NPV of MRI-guided biopsy for diagnosing a pCR was 92.8% (95% CI, 66.2%-99.8%) when pCR definition 1 was used and 85.8% (95% CI, 57.2%-98.1%) when pCR definition 2 was used. When pCR definition 1 was used, there was 1 false-negative MRI-guided biopsy result for which surgical pathology findings demonstrated less than 0.02 cm of residual invasive disease. This patient’s pre-NAC tumor measured 5.4 cm, which is larger than the median tumor size of 3.0 cm. When pCR definition 2 was used, there was a second false-negative MRI-guided biopsy result for which surgical pathology findings demonstrated residual ductal carcinoma in situ. This patient’s pre-NAC tumor measured 2.4 cm, which also is smaller than the median.

**Table 2.** Breast Cancer NAC Response and Accuracy of MRI-Guided Biopsy Results for Diagnosing a pCR

	Definition 1^a^	Definition 2^a^
No. (%) of patients		
pCR	13 (65)	12 (60)
No pCR	7 (35)	8 (40)
Parameter, estimate, % (95% CI)		
NPV	92.8 (66.2-99.8)	85.8 (57.2-98.1)
Specificity	100 (NA)	100 (NA)
PPV	100 (NA)	100 (NA)
Sensitivity	85.8 (42.0-99.6)	75.0 (35.0-96.8)
Accuracy	95.0 (75.1-99.9)	90.0 (68.3-98.7)

Abbreviations: MRI, magnetic resonance imaging; NA, not applicable; NPV, negative predictive value; pCR, pathologic complete response; PPV, positive predictive value.

^a^ Definition 1 defined a pCR as no residual invasive cancer; definition 2 defined a pCR as no residual invasive or in situ cancer.

Logistic regression showed that age was not significantly associated with surgical pathology findings using pCR definition 1 (log odds ratio (OR), −0.01; 95% CI, −0.14 to 0.12; *P* = .90) or pCR definition 2 (log OR, −0.01; 95% CI, −0.13 to 0.12; *P* = .92). Breast cancer subtype was also not significantly associated with surgical pathology findings using pCR definition 1 (for hormone receptor (HR)–positive, *ERBB2*-negative cancer: log OR, 0.29; 95% CI, −3.91 to 4.49; *P* = .89; for HR-negative, *ERBB2*-positive cancer: log OR, −0.14; 95% CI, −4.27 to 3.99; *P* = .95; for triple-negative cancer: log OR, 0.86; 95% CI, −2.35 to 4.07; *P* = .60) or pCR definition 2 (for HR-positive, *ERBB2*-negative cancer: log OR, 2.94; 95% CI, −2.19 to 8.07; *P* = .26; for HR-negative, *ERBB2*-positive cancer: log OR, −0.23; 95% CI, −4.37 to 3.90; *P* = .91; for triple-negative cancer: log OR, 0.77; 95% CI, −2.42 to 3.95; *P* = .64). However, the MRI-guided biopsy findings were positively associated with surgical pathology data using both pCR definition 1 (log OR, 3.69; 95% CI, 0.35 to 7.04; *P* = .03) and pCR definition 2 (log OR, 3.51; 95% CI, −0.03 to 7.06; *P* = .05) (eFigure in Supplement 2). Last, correlation analysis using the Kendall rank correlation coefficient showed a positive association between MRI-guided biopsy results and surgical resection pathology findings for both definitions of pCR (τ = 0.80; *P* < .001).

## Discussion

These preliminary results suggest that MRI-guided biopsy was an accurate approach for evaluating the extent of residual disease after NAC. Of the 2 patients with a false-negative MRI-guided biopsy result for definition 2 pCR, 1 had a focus of ductal carcinoma in situ and the other had a microscopic focus of residual invasive cancer. Hence, these findings suggest that a negative MRI-guided biopsy result was a reliable indicator of pCR, or of limited residual disease, after NAC. Surgery has historically been the mainstay for breast tissue diagnosis. Beginning in 1995, we reported that image-guided breast interventions can mimic the accuracy of diagnostic surgical procedures.^19^ The accuracy of MRI-guided biopsy was subsequently demonstrated by our group in 2003,^17,20^ and has now become a standard-of-care approach. Building on these developments, we hypothesized that MRI-guided biopsy might represent an accurate image-guided approach to diagnosing a pCR, potentially obviating the need for surgical resection among patients with no evidence of residual tumor. This image-based approach could potentially represent a minimally invasive alternative to a surgical procedure of unclear benefit.

In the literature, other studies^21,22,23,24^ reported high false-negative rates (FNRs), which represent an incorrect biopsy pCR that is pathologically a non-pCR. Heil et al^21^ included all biological subtypes with a partial or complete imaging response and reported an FNR of 17.8% (n = 398) with vacuum-assisted biopsy performed under either ultrasonographic or mammographic guidance. Tasoulis et al^22^ reported an FNR of 18.7% (n = 166) with image-guided vacuum-assisted biopsy after NAC, which was performed in 86% of patients. Basik et al^23^ included near-complete or radiologically complete response by triple-modality imaging (defined on MRI as no mass, with rapid rise or washout kinetics) and reported that the NRG Oncology BR005 trial^25^ had an FNR of 50% (n = 98) with stereotactic core needle biopsy as well as an NPV of biopsy results for pCR of 77.5%. Vrancken-Peeters et al^24^ included partially or radiologically complete response on MRI and reported that the Minimally Invasive Complete Response Assessment (MICRA) trial^26^ had an interim FNR of 37% (n = 167) with ultrasound core needle biopsy and an interim NPV of biopsy results for pCR of 75.4%. The NRG Oncology BR005 trial^25^ and the MICRA trial^26^ both used post-NAC MRI to identify eligible patients, but MRI-guided biopsy was not performed. Vrancken-Peeters et al^24^ also reported that vacuum-assisted biopsy had a lower FNR than core needle biopsy. Rauch et al^14^ reported the highest accuracy of image-guided biopsy, with an FNR of 0% (n = 40) for vacuum-assisted biopsy performed by either stereotaxis (63%) or ultrasonography (37%), but they also reported that the ultrasound-guided biopsy subgroup analysis demonstrated an FNR of 40% and a PPV of the biopsy result for pCR of 60%. We were unable to reconcile this discrepancy on review of their published data.^27^ Other studies are currently being conducted, including the NOSTRA-Feasibility Study.^28^ Finally, at present, studies of a putative nonoperative paradigm are being conducted by the MD Anderson Cancer Center,^29^ whereas others are in the planning stages. However, none of these studies have incorporated MRI-guided biopsy to diagnose a pCR, which, to our knowledge, represents the most sensitive imaged-based approach to date.

### Limitations

Our study has a few limitations. Our findings must be interpreted in the context of the trial design. As a pilot study, analyses are limited by the small cohort size that established feasibility. Moreover, our methodology was entirely dependent on advanced breast MRI imaging approaches; extrapolation of our results to other imaging modalities must be made with caution.

## Conclusions

We present herein the results of a feasibility study that suggest the potential utility of MRI-guided biopsy to identify pCR after NAC. This nonrandomized controlled trial’s results suggest that the accuracy of MRI-guided biopsy to diagnose a post-NAC pCR approaches that of reference-standard surgical resection in this small cohort. With an increasing focus on mitigating overtreatment and limiting potential toxic effects and cost, MRI-guided biopsy may be a viable alternative to surgical resection for appropriately selected patients after NAC. These preliminary results support the need for further study of our novel method that uses MRI-guided biopsy.

## Notes

Supplement 1. **Trial Protocol**

Supplement 2. **eFigure.** ROC Curve for the Logistic Regression Model to Measure the Diagnostic Accuracy of MRI Biopsy in Detecting pCR

Supplement 3. **Data Sharing Statement**
