# Supplementary material for: Accuracy of Magnetic Resonance Imaging–Guided Biopsy to Verify Breast Cancer Pathologic Complete Response After Neoadjuvant Chemotherapy: A Nonrandomized Controlled Trial
Source: JAMA Netw Open. 2021 Jan 15;4(1):e2034045. doi: 10.1001/jamanetworkopen.2020.34045 (PMC7811182; doi:10.1001/jamanetworkopen.2020.34045)
Supplement: Supplement 1. — Trial Protocol [file jamanetwopen-e2034045-s001.pdf]

A Prospective Study To Evaluate The Accuracy Of Percutaneous Breast MRI Biopsy In Diagnosing A Pathologic Complete Response Following Neoadjuvant Chemotherapy

PROTOCOL FACE PAGE FOR
MSK NON THERAPEUTIC PROTOCOL

Principal Investigator/Department:	Elizabeth J. Sutton, MD	Radiology
Co-Principal Investigator(s)/Department:	Mahmoud El-Tamer, MD<br>Virgilio Sacchini, MD	Surgery<br>Surgery
Investigator(s)/Department:	Elizabeth Morris, MD<br>Janice Sung, MD<br>Maxine Jochelson, MD<br>Andrea Abramson, MD<br>Yolanda Bryce, MD<br>Donna D'Alessio, MD<br>Kimberly N. Feigin, MD<br>Mary Hughes, MD<br>Delia Keating, MD<br>Carol Lee, MD<br>Christopher E. Comstock, MD<br>Sandra Brennan, MD<br>Betty Ann Caravella, MD<br>Stefanie Jacobs, MD<br>Jill S. Gluskin, MD<br>Tammy Huang, MD<br>Jennifer Kaplan, MD<br>Lizza Lebron-Zapata, MD<br>Christoper Riedl, MD<br>Victoria L. Mango, MD<br>Katja Pinker-Domenig, MD<br>Jeffrey Reiner, MD<br>Nicole Saphier, MD<br>Katherine Gallagher, MD<br>Meredith Sadinski, PhD<br>Lori Kelly, MD<br>Jung Hun Oh, PhD<br>Aditya Apte, PhD<br>Harini Veeraraghavan, PhD<br>Pedram Razavi, MD<br>Mithat Gonen, PhD<br>Marinela Capanu, PhD<br>Jorge Reis-Filho, MD, PhD<br>Edi Brogi, MD, PhD<br>Lior Braunstein, MD<br>Andrea V. Barrio, MD<br>Deborah M. Capko, MD<br>Hiram S. Cody III, MD<br>Mary L. Gemignani, MD	Radiology<br>RadiologyMedical Physics<br>Medical Physics<br>Medical Physics<br>Graduate Medical Education<br>Epidemiology/Biostatistics<br>Epidemiology/Biostatistics<br>Pathology<br>Pathology<br>Radiation Oncology<br>Surgery<br>Surgery<br>Surgery<br>Surgery

	Laurie J. Kirstein, MD<br>Monica Morrow, MD<br>Melissa L. Pilewskie, MD<br>George Plitas, MD<br>Lisa M. Sclafani, MD<br>Kimberly J. Van Zee, MD<br>Alexandra S. Heerdt, MD<br>Tracy-Ann S. Moo, MD<br>Audree Tadros, MD	Surgery<br>Surgery<br>Surgery<br>Surgery<br>Surgery<br>Surgery<br>Surgery<br>Surgery<br>Surgery
Consenting Professional(s)/Department:	Elizabeth Sutton, MD<br>Elizabeth Morris, MD<br>Andrea Abramson, MD<br>Sandra Brennan, MD<br>Yolanda Bryce, MD<br>Betty Ann Caravella, MD<br>Christopher Comstock, MD<br>Donna D'Alessio, MD<br>Kimberly Feigin, MD<br>Jill Gluskin, MD<br>Tammy Huang, MD<br>Mary C. Hughes, MD<br>Stefanie Jacobs, MD<br>Maxine Jochelson, MD<br>Jennifer Kaplan, MD<br>Delia Keating, MD<br>Lori Kelly, MD<br>Lizza Lebron-Zapata, MD<br>Carol Lee, MD<br>Victoria Mango, MD<br>Katja Pinker-Domenig, MD<br>Jeffrey Reiner, MD<br>Nicole Saphier, MD<br>Janice Sung, MD<br>Katherine Gallagher, MD<br>Mahmoud El-Tamer, MD<br>Virgilio Sacchini, MD<br>Andrea V. Barrio, MD<br>Deborah M. Capko, MD<br>Hiram S. Cody III, MD<br>Mary L. Gemignani, MD<br>Alexandra Heerdt, MD<br>Laurie J. Kirstein, MD<br>Monica Morrow, MD<br>Melissa L. Pilewskie, MD<br>George Plitas, MD<br>Lisa M. Sclafani, MD<br>Kimberly J. Van Zee, MD<br>Tracy-Ann S. Moo, MD<br>Audree Tadros, MD	Radiology<br>Surgery<br>Surgery<br>Surgery<br>Surgery<br>Surgery<br>Surgery<br>Surgery<br>Surgery<br>Surgery<br>Surgery<br>Surgery<br>Surgery<br>Surgery<br>Surgery<br>Surgery

Please Note: A Consenting Professional must have completed the mandatory Human Subjects Education and Certification Program.

OneMSK Sites	
Manhattan	All Protocol Activities
Basking Ridge	Consent Only
Commack	Consent Only
Westchester	Consent Only
Monmouth	Consent Only
Bergen	Consent Only

Table of Contents

1.0 PROTOCOL SUMMARY AND/OR SCHEMA 5
2.0 OBJECTIVES AND SCIENTIFIC AIMS 7
3.0 BACKGROUND AND RATIONALE 8
4.0 OVERVIEW OF STUDY DESIGN/INTERVENTION 10
4.1 Design 10
4.2 Intervention 11
5.0 CRITERIA FOR SUBJECT ELIGIBILITY 12
5.1 Subject Inclusion Criteria 12
5.2 Subject Exclusion Criteria 12
6.0 RECRUITMENT PLAN 13
7.0 ASSESSMENT/EVALUATION PLAN 14
8.0 TOXICITIES/SIDE EFFECTS 15
9.0 PRIMARY OUTCOMES 16
10.0 CRITERIA FOR REMOVAL FROM STUDY 16
11.0 BIOSTATISTICS 17
12.0 RESEARCH PARTICIPANT REGISTRATION AND RANDOMIZATION PROCEDURES 18
12.1 Research Participant Registration 18
Confirm eligibility as defined in the section entitled Criteria for Patient/Subject Eligibility. 18
12.2 Randomization 18
13.0 DATA MANAGEMENT ISSUES 18
13.1 Quality Assurance 18
13.2 Data and Safety Monitoring 19
14.0 PROTECTION OF HUMAN SUBJECTS 19
14.1 Privacy 20
14.2 Serious Adverse Event (SAE) Reporting 20
14.2.1 21
15.0 INFORMED CONSENT PROCEDURES 21
16.0 REFERENCES 23
17.0 APPENDICES 24

1.0 PROTOCOL SUMMARY AND/OR SCHEMA

Targeted systemic therapy, based upon a tumors molecular subtype, in the neoadjuvant chemotherapy (NAC) setting epitomizes the concept of precision medicine, which we would like to take one step further. This research project is set to challenge the current clinical practice that all women who undergo NAC must still undergo surgery. We hypothesize that in a certain subset of women who have had a complete imaging response to NAC that pathology from a percutaneous magnetic resonance imaging (MRI) guided biopsy will accurately diagnose a pathologic complete response (pCR) therefore obviating the need for breast surgery (Figure 1). This novel method challenges the current clinical practice and may lead to a paradigm shift in treatment practices and the meaning of breast conservation therapy. The scientific and clinical impact of this investigation will be far reaching. Our hypothesis challenges the status quo and surgical techniques that have not significantly changed since the 1980s. In the short term, we hope that this first-in-human clinical trial on HER2 overexpressing, ER positive or triple negative breast cancers will establish feasibility and ultimately allow the technique to be investigated in all breast cancer molecular subtypes and ultimately become a new and equally effective minimally invasive way of treating breast cancer. Further, this procedure will be cost effective and will save a tremendous amount of money over time since a percutaneous biopsy is significantly less expensive than surgery. In the longer term and broader context, we believe that this has the potential to be a major paradigm shift in the clinical treatment of what is currently often referred to as “operable breast cancer”. We believe that this is the future of breast cancer precision medicine. Most importantly, this work has the potential to impact all younger generations ultimately offering the possibility of saving their breasts—to many, the hallmark and essence of femininity.

This prospective single institution pilot study will compare percutaneous breast MRI biopsy following NAC to reference-standard breast surgery specimen in diagnosing pCR in 25 patients with either a HER2 overexpressing, ER positive or triple negative operable breast cancers. We anticipate an average accrual rate of 1 patient per month with a complete accrual of 25 patients within 24 months.

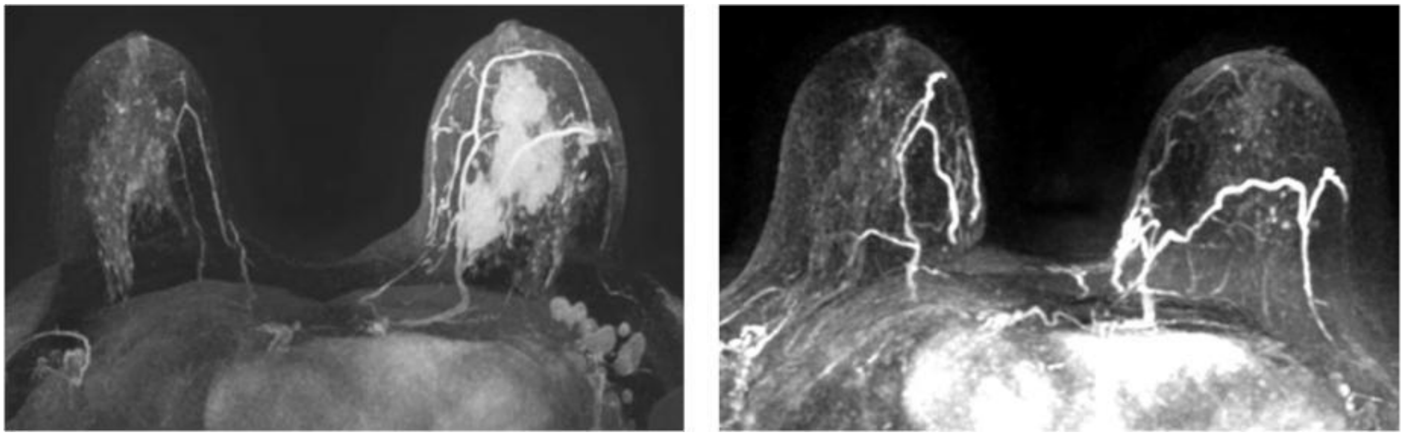

Figure 1: Patient with left breast HER2 overexpressing breast cancer (arrow) pre (A) and post (B) NAC that underwent mastectomy and had a pCR, no residual cancer in the surgically removed breast.

The study schema as follows:

At First Visit for Potential Study Candidates In Surgery Clinic Post NAC and MRI

- Post treatment MRI results are reviewed and potential candidate is confirmed to have operable breast cancer
- Non-study lumpectomy or mastectomy date is scheduled
- Informed consent is provided

↓

Percutaneous Biopsy Eligibility

- Potential study candidates evaluated for eligibility
- If eligible then:
  - Informed consent signed
  - MRI core biopsy date will be set for 0-60 days after completing NAC and before surgery

↓

Percutaneous MRI Biopsy

- MRI examinations will be performed (depending upon schedule availability) on either at 1.5T or 3.0T whoele-body MRI unit (GE Medical Systems, Waukesha, WI) equipped with either a dedicated 8 or 16-channel surface breast coil
- The MRI biopsy will be performed without the administration of intravenous contrast

↓

Standard of Care Surgery

↓

Compare MRI Biopsy With Final Surgery Pathology

Standard of care NAC protocol:

HER 2 overexpressing breast cancers:

All patients with HER2 overexpressing breast cancer will be treated with the same standard-of-care systemic NAC regimen at MSK, AC-THP (Doxorubicin-cyclophosphomide-paclitaxel-traztuzumab-pertuzumab). The MSK regimen is AC (Doxorubicin-cyclophosphomide) given every 2 weeks for four cycles; followed by Paclitaxel (T) given every 2 weeks for four cycles surgery is performed after the last cycle of Paclitaxel). HP (traztuzumab-pertuzumab) is started concurrently with Paclitaxel and given for 1 year.

Triple negative breast cancers:

All patients with triple negative breast cancer will be treated with the same standard-of-care systemic NAC regimen at MSK, AC-T (Doxorubicin-cyclophosphomide-paclitaxel). The MSK regimen is AC (Doxorubicin-cyclophosphomide) given every 2 weeks for four cycles; followed by Paclitaxel (T) given every 2 weeks for four cycles (surgery is performed after the last cycle of Paclitaxel).

Standard of care clinical Breast MRI:

All patients will undergo standard-of-care clinical bilateral breast MRI at two time points, pre and post NAC. All post NAC MRI will take place after the last cycle of Paclitaxel, which is approximately 16 weeks after the pre NAC MRI. The pre and post NAC MRI examinations will be performed (depending upon schedule availability) on either a 1.5T or 3.0-T whole-body MRI unit (GE Medical Systems, Waukesha, WI) equipped with either a dedicated 8 or 16-channel surface breast coil. All sequence parameters will be similar. The pre and post NAC clinical breast MRI will be interpreted by a Breast Imaging radiologist according to the Breast Imaging-Reporting and Data System (BI-RADS) Lexicon.

2.0 OBJECTIVES AND SCIENTIFIC AIMS

Primary Objective:

Compare percutaneous breast MRI biopsy following neoadjuvant chemotherapy (NAC) to reference-standard breast surgery specimen in diagnosing pathologic complete response (pCR) in HER2 overexpressing, ER positive or triple negative operable breast cancer patients with a complete imaging response. We are primarily interested in estimating the negative predictive value of MRI biopsy: probability that a biopsy negative patient has a complete pathological response.

Secondary Objective:

Evaluate the other metrics of accuracy (sensitivity, specificity, positive predictive value) of MRI in diagnosing a complete pathological response post-NAC.

3.0 BACKGROUND AND RATIONALE

This research project, utilizing imaging, will be the first to challenge the current clinical practice that all women who undergo NAC for operable breast cancer must still undergo surgery.

Breast cancer is the second leading cause of cancer deaths among women in the United States and in 2015 there were approximately 234,190 new cases and 40,730 deaths[1]. Thus it remains a major public health problem. In the literature, it is also increasingly established that not all breast cancers are the same and tumor behavior appears to vary according to differences in genetic molecular subtypes[2]. The proposed research project takes into account the major shift in the treatment of operable breast cancer involving neoadjuvant chemotherapy (NAC) and the differences in genetic molecular subtypes. NAC is the administration of chemotherapy prior to definitive breast cancer surgery [3]. NAC can enable breast-conserving surgery and sentinel lymph node biopsy in women who traditionally required a mastectomy and full axillary lymph node dissection [3, 4]. The primary goal of NAC is a pathologic complete response (pCR) defined as the absence of any residual cancer (both invasive and in situe carcinoma)[5]. As a biomarker, pCR serves as an intermediate endpoint for improved disease free and overall survival[5, 6]. Recent studies have shown that breast cancer response to NAC is based upon its molecular subtype with human epidermal growth factor receptor 2 (HER2) overexpressing, ER positive and triple negative invasive ductal carcinomas having the highest likelihood of pCR, up to 45.4% and 38.2% respectively[3, 7].

Imaging is critical in the evaluation of individual treatment response to NAC to determine if a patient is a candidate for breast conservation therapy. The National Comprehensive Cancer Network guidelines currently recommend a breast MRI pre and post NAC. Numerous studies have demonstrated that MRI has the highest predictive value for pCR which was reported to be 73.6% in a cohort of patients with HER2 overexpressing, ER positive and triple negative breast cancers [3, 8.] This research project, utilizing imaging, will be the first to challenge the current clinical practice that all women who undergo NAC for operable breast cancer must still undergo surgery.

Breast cancer encompasses a heterogeneous group of tumors with very different outcomes. Therefore, gene expression patterns have been used to define tumor molecular subtypes that are both prognostic and predictive of treatment response[2, 9]. Cancer whole-genome sequencing is not yet the clinical standard so currently immunohistochemical surrogates are used to define an individual patient’s breast cancer molecular subtype. Targeted systemic therapy, based upon a tumor’s molecular subtype, in the NAC setting epitomizes the concept of precision medicine, which we would like to take one step further. MRI has the highest diagnostic accuracy for pCR [3, 8] following NAC. However, it is not 100%; therefore, all women still undergo breast surgery. A significant number of patients with HER2 overexpressing (45.4%) and triple negative (38.2%) breast cancers post NAC will find out from the pathology report that there was no residual breast cancer in their already surgically removed breast[3,7]. This leaves some women very upset, confused, and wondering why they had to have any surgery if there was no cancer left.

We have decided to limit our proof-of-concept study to HER2 overexpressing, ER positive and triple negative breast cancers being treated with NAC. As mentioned above, they have the highest likelihood of pathologic complete response. At MSK, each tumor molecular subtype (as defined using immunohistochemical surrogates) are uniformly treated with the same systemic therapy. HER2 overexpressing tumors represent approximately 15-20% and triple negative tumors represent approximately 15-20% of all new breast cancer patients at our institution[3, 7, 10]. HER2 overexpression is detected by immunohistochemistry (protein expression) and/or fluorescence in situ hybridization (gene amplification)[11]. HER2, a membrane tyrosine kinase receptor, is a proto-oncogene and belongs to the epidermal growth factor receptor (EGFR) family and can activate different cellular pathways responsible for cell differentiation, growth and survival including the p44/42 mitogen-activated protein kinase (MAPK) and phosphatidylinositol-3-kinase (PI3K) pathways[12-14].

Analogous to how the Breast Imaging Service at MSKCC were pioneers in proving that a percutaneous breast biopsy could replace a surgical excisional biopsy in most settings, we hope to prove that a percutaneous breast biopsy can replace surgery for some women post NAC who have had a complete imaging response. The scientific and clinical impact of this investigation will be far reaching. Our hypothesis challenges the status quo and surgical techniques that have not significantly changed since the 1980s. In the short term, we hope that this first in-human clinical trial on HER2 overexpressing, ER positive and triple negative breast cancers will establish feasibility and ultimately allow the technique to be investigated in all breast cancer molecular subtypes and ultimately become a new and equally effective minimally invasive way of treating breast cancer. Further, this procedure will be cost effective and will save a tremendous amount of money over time since a percutaneous biopsy is significantly less expensive than surgery. In the longer term and broader context, we believe that this has the potential to be a major paradigm shift in the clinical treatment of what is currently often referred to as “operable breast cancer.” We believe that this is the future of breast cancer precision medicine: high resolution MR imaging, targeted systemic therapy, and a minimally invasive percutaneous biopsy with or without adjuvant radiation therapy. This work could have a transformational effect suggesting that breast cancer in the future will be a non-surgical disease. Most importantly, this work has the potential to impact all younger generations ultimately offering the possibility of saving their breasts–to many, the hallmark and essence of femininity. In terms of innovation, we will be the first institution to perform this in-human clinical trial and if successful this scientific knowledge will allow us to expand it to include all breast cancer molecular subtypes being treated with NAC. This will also be the first study to suggest a non-surgical alternative treatment for early stage breast cancer post NAC.

4.0 OVERVIEW OF STUDY DESIGN/INTERVENTION

4.1 Design

Primary Objective: Compare percutaneous breast MRI biopsy following neoadjuvant chemotherapy (NAC) to reference-standard breast surgery specimen in diagnosing pathologic complete response (pCR) in HER2 overexpressing, ER positive or triple negative breast cancers with a complete imaging response. We are primarily interested in estimating the negative predictive value of MRI biopsy: probability that a biopsy negative patient has a complete pathological response.

This is a pilot prospective single institution study that will enroll 25 women who: (a) underwent a pre and post NAC clinical breast MRI for histologically confirmed HER2 overexpressing, ER positive or triple negative invasive ductal or invasive lobular breast cancer; (b) had a complete imaging response to treatment on the post NAC clinical breast MRI defined as no residual tumor enhancement and (c) will undergo definitive breast surgery at our institution.

Consenting, eligible women will then be assigned a date for MRI-guided biopsy 0-60 days after completing NAC and before surgery. The study intervention will be defined by the pre-determined, non-study surgical date. Research study intervention will not interfere with the planned, standard-of-care, surgery arrangements.

Percutaneous biopsy: All patients enrolled will have had complete MR imaging response post NAC and will undergo percutaneous MR guided biopsy. All MR guided biopsies will be performed with either a 1.5-T or 3.0T whole-body MRI GE unit equipped with a dedicated 8- or 16-channel surface breast coil. Because the patients have had a complete imaging response to NAC, which means there is no residual tumor enhancement, this biopsy will be performed without intravenous contrast. Our biopsy protocol will include a localizing sequence followed by acquisition of sagittal T1-weighted, T1-weighted fat-suppressed and T2-weighted fat-suppressed images without intravenous contrast. Biopsies will be performed with a 9-guage vacuum-assisted MRI compatible biopsy system (ATEC Breast Biopsy System, Suros Surgical Systems, Indianapolis, IN). Biopsy target will be the treated tumor bed defined by the accurately positioned pre NAC biopsy marker (this is the standard of care biopsy, which may have been done under stereotactic, ultrasound or MRI guided biopsy) and/or anatomic landmarks. 12 samples will be taken through one incision site and sent to pathology for analysis. A titanium marker will be placed post-biopsy under MRI guidance and a post-biopsy mammogram will be performed to document adequate positioning.

Pathologic analysis*:* The percutaneous biopsy pathology and surgical specimen will be evaluated for chemotherapeutic response. A pCR is defined as absence of invasive and in situ tumor cells. Miller-Payne will be used to assess the tumor response.

1-Percutaneous biopsy: Two reference breast pathologists will independently evaluate the biopsy specimens. If there is discordance between these two interpretations an additional review will be performed by a third.

2-*Surgical specimen*: This will be evaluated, per standard clinical practice, by a breast pathologist, blinded to the pathology results of the percutaneous MRI biopsy, and results will be collected retrospectively from the final surgical pathology report.

4.2 Intervention

The MRI-guided biopsies will be performed by investigators from the Breast Radiology Service. The research MRI guided percutaneous biopsy technique will be similar to that performed for clinical diagnosis except it will not require the administration of intravenous contrast because the patients tumor has had a complete imaging response as documented on the post-treatment clinical bilateral breast MRI as defined by the absence of tumor enhancement. All MR guided biopsies will be performed with either a 1.5-T or 3.0T whole-body MRI GE unit equipped with a dedicated 8- or 16-channel surface breast coil. On the day of the procedure, the site of biopsy proven cancer will first be localized with MRI using the accurately position pre-NAC biopsy marker as well as anatomic landmarks to define the treated tumor bed. Our biopsy protocol will include a localizing sequence followed by acquisition of sagittal T1-weighted, T1-weighted fat-suppressed and T2-weighted fat-suppressed images.

Biopsies will be performed with a 9-guage vacuum-assisted MRI compatible biopsy system (ATEC Breast Biopsy System, Suros Surgical Systems, Indianapolis, IN). The skin will be cleansed with Betadine and 3 cc of 1% lidocaine will be used for superficial anesthesia. 10 cc of 1% lidocaine with epinephrine (1:200,000; 5mcg/ml) will be used for deep anesthesia. A skin nick will be made. The 9-gauge vacuum-assisted MRI compatible biopsy device will be inserted when target confirmed through the single skin nick and directed to the region of biopsy proven cancer. 12 core biopsy specimens will then be obtained at the same time through a vacuum. After MRI guided biopsy is complete, a titanium marker will be placed at the site of the MRI guided biopsy. The specimens will be dropped off to pathology for analysis.

Following the procedure, pressure will be held on the site(s) until bleeding ceases. The area(s) will be cleansed and Steri-Strips applied. Post biopsy instructions will be given verbally and in writing. Risks of the procedure include pain, bleeding and infection. The non-therapeutic intervention will not impact date or type of breast surgery.

A post-biopsy mammogram will be performed to document adequate position of the titanium marker within the MRI guided biopsy cavity. If the post-treatment MRI guided biopsy titanium marker has been displaced, a second titanium biopsy marker can be placed immediately in real time under ultrasound guidance if the post-treatment MRI guided biopsy displaced titanium marker would impact standard clinical care surgery as determined by the patient's surgeon.

The research percutaneous biopsy specimens will undergo standard of care pathology analysis and will be evaluated by two reference breast pathologists independently. If there is discordance between these two interpretations an additional review will be performed by a third.

At the time of breast surgery, pathologic complete response will be determined by standard department of pathology methods and the amount of residual cancer burden will be estimated by a pathologist, who will be blinded to the pathology of the percutaneous MRI biopsy.

If a patient experiences side effects from intervention resulting in a delay of their planned surgery date, this will be considered a complication. The planned intervention is to perform MR guided biopsy post-NAC in the region of the index tumor.

5.0 CRITERIA FOR SUBJECT ELIGIBILITY

5.1 Subject Inclusion Criteria

Subjects must meet the following criteria at screening to be eligible to participate in the study:

- Women age 18 years or older
- Confirmed histologic diagnosis of operable HER2 overexpressing (ER<10%, PR<10%, and HER2 2+ or FISH amplified) OR triple negative (ER<10%, PR<10%, and HER2 0/1+ or 2+/FISH not amplified) OR ER positive invasive ductal or invasive lobular breast cancer, including MSKCC pathology confirmation
- Operable breast cancer treated with NAC undergoing either breast conservation or total Mastectomy who have had a post-NAC clinical bilateral breast MRI demonstrating a complete imaging response, which is defined as no residual tumor enhancement.
- No indication of distant metastases (M0)
- Tumor site amenable to MRI guided biopsy as determined by the radiologist
- Definitive surgery being performed at MSKCC within within 0-60 days of completing NAC
- ECOG performance status score of 0 to ≤2
- Women of childbearing potential (WOCBP) must not be pregnant.
- Women must not be breastfeeding
- Willing and able to provide informed consent and adhere to the study visit schedule and plan as specified in this protocol

5.2 Subject Exclusion Criteria

- Medical history and concurrent disease:
    - Prior history of treated breast cancer
    - Any underlying medical or psychiatric conditions, which in the opinion of the investigator, will make performing the study intervention hazardous or obscure the interpretation of the results
- Prohibited Treatments and/or Therapies:
    - Prior history of breast cancer surgery and/or radiotherapy.

6.0 RECRUITMENT PLAN

These aims were designed because at Memorial Sloan Kettering Cancer Center (MSKCC) we see over 2000 new breast cancer diagnoses each year of which approximately 400 undergo NAC and HER2 overexpressing and triple negative invasive breast cancers each account for approximately 15-20% of our population. We anticipate an average accrual rate of 0-1 patients per month with a complete accrual of 25 patients within 24 months.

Electronic medical records will be reviewed by the clinical research team to determine patient eligibility. As soon as possible, potentially eligible patients will be evaluated by the principal investigator (Dr. Elizabeth Sutton). At the time of the post-NAC pre-surgical appointment, potentially eligible patients will be identified and surgery-related dates requested/set per standard-of-care.

A consenting professional listed on the study will consent interested patients in a private clinical space to our prospective IRB approved protocol between the post-NAC pre-surgical visit and the day of the MRI biopsy. All patients who are recruited will be added to this database. Clinical data, including current treatment, will be updated on each patient within the database on a weekly basis. MSK has filed Form HHS441 (Re: Civil Rights) and form HHS 641 (Re: Handicapped Individuals). In selecting patients for study in the proposed project, no exclusions are or will be made on ethnicity, race or age.

At first visit for potential study candidates in surgery clinic post NAC and MRI

- Post-treatment MRI results are reviewed and candidate confirmed to have operable breast cancer
- Non-study lumpectomy or mastectomy date is scheduled
- Informed consent will be obtained between this visit and the day of the MRI biopsy.
- MRI biopsy is scheduled to occur as soon as possible

Patients will be recruited for enrollment on this trial primarily through referrals from their primary surgeon. The clinical trial will be listed on the clinicaltrials.gov website as well as on the MSKCC website. Patients will not be paid for participation in this study. Prior to enrollment on the study, the physician will discuss the study protocol in detail with the patient, including possible complications.

Potential research subjects will be identified by a member of the patient’s treatment team, the protocol investigator, or Breast Radiology research team at Memorial Sloan Kettering Cancer Center (MSKCC). If the investigator is a member of the treatment team, she/he will screen their patient’s medical records for suitable research study participants and discuss the study and their potential for enrolling in the research study. Potential subjects contacted by their treating physician will be referred to the investigator/research staff of the study.

The principal investigator and Breast Radiology research team may also screen the medical records of patients with whom they do not have a treatment relationship for the limited purpose of identifying patients who would be eligible to enroll in the study and to record appropriate contact information in order to approach these patients regarding the possibility of enrolling in the study.

Eligible patients will be either contacted by telephone or approached on the day of their post-NAC pre-surgical appointment to discuss the study briefly and to see if they would be willing to participate. Consent will not be obtained over the telephone. A consenting professional listed on the study will consent interested patients within a private clinical space between the first visit in the surgery clinic post NAC and MRI and the of the MRI biopsy.

During the initial conversation between the investigator/research staff and the patient, the patient may be asked to provide certain health information that is necessary to the recruitment and enrollment process. The investigator/research staff may also review portions of their medical records at MSKCC in order to further assess eligibility. They will use the information provided by the patient and/or medical record to confirm that the patient is eligible and to contact the patient regarding study enrollment. If the patient turns out to be ineligible for the research study, the research staff will destroy all information collected on the patient during the initial conversation and medical records review, except for any information that must be maintained for screening log purposes.

In most cases, the initial contact with the prospective subject will be conducted either by the treatment team, investigator or the research staff working in consultation with the treatment team. The recruitment process outlined presents no more than minimal risk to the privacy of the patients who are screened and minimal PHI will be maintained as part of a screening log. For these reasons, we seek a (partial) limited waiver of authorization for the purposes of (1) reviewing medical records to identify potential research subjects and obtain information relevant to the enrollment process; (2) conversing with patients regarding possible enrollment; (3) handling of PHI contained within those records and provided by the potential subjects; and (4) maintaining information in a screening log of patients approached (if applicable).

7.0 ASSESSMENT/EVALUATION PLAN

Screening: The following must be completed within 28 days of study start

- Signed protocol informed consent
- Medical history
- Medication list review
- ECOG performance status
- Pathology confirmed at MSKCC. Local assessment of hormone receptor and HER2 status acceptable for study participation.
- Neoadjuvant chemotherapy completed
- Post-treatment MRI reviewed

After the patient is identified as a potentially appropriate study candidate at the time of breast surgery appointment post neoadjuvant chemotherapy and MRI, the principal investigator (Dr. Elizabeth Sutton) will confirm eligibility and then one of the consenting professionals will review informed consent with study candidate including potential complications. Eligibility for safe MRI guided biopsy will include an assessment of how posterior the site of index tumor is because if it

is less than 1 cm from the pectoralis muscle this may preclude our ability to safely biopsy it. If the site of the patient's index tumor is amenable to MRI guided biopsy and informed consent has been signed, the patient will be scheduled for the biopsy.

No delay in surgery will be made to accommodate participation in the protocol.

Day 1 (must be on a Monday through Friday):

- Percutaneous MRI guided biopsy by study Breast Radiologist within 0-60 days of completing NAC.
- Post-biopsy mammogram performed.
- Pre-operative toxicity review.
- Proceed to definitive surgery within within 0-60 days of completing NAC.

Post-operative Surgery visit: The pathology of the percutaneous MRI biopsy will be documented and compared to the pathology results at the time of surgery.

	Screening	Day 0-60	After planned biopsy
Signed informed consent	X		
Medical history	X		
Medication review	X		
ECOG PS	X		
Pathology confirmed at MSKCC	X		
Neoadujuvant chemotherapy protocol completed and confirmed at MSKCC	X		
Pre and post-treatment MRI reviewed at MSKCC	X		
Research MRI guided biopsy in the region of breast tumor		X	
Pathology analysis of percutaneous biopsy			X
Definative breast cancer surgery			X
Pathology analysis of surgical specimen			X
Protocol review			X

8.0 TOXICITIES/SIDE EFFECTS

Due to MRI, patients will be counseled regarding the MRI biopsy, which is an FDA approved technique. Breast MRI biopsy is currently being offered to any patient that needs it at MSK both for high risk screening and in the context of a new diagnosis of breast cancer. The use of magnets to obtain chemical information has been studied previously with minimal side effects. As is the case for a standard MR exam, the presence of a pacemaker or aneurysm clips could pose a risk

to the patient and patients with these contraindications will be excluded. A subject with sensitivity to noise or some neurological problems may have difficulty tolerating the MRI noise. Therefore it is recommended that patients talk to their referring physician regarding the noise of the MRI prior to taking part in the study. Some patients feel claustrophobic (afraid of enclosed spaces) in the MRI magnet.

Rare but Serious due to MRI:

- Another possible hazard of the exam is localized heating of the body due to the radio waves used. If this were to happen, the patient would feel an intense heating or burning sensation. The patient will be asked to notify the MR technologist immediately. However, the MR scanner and the MR cool has been designed to prevent this from happening and there have been no reports of local heating in patients scanned to date.
- Because the MR instrument attracts iron, any iron-containing objects will accidentally fly into the magnet causing injury. Precautions have been made to prevent this from happening.
- The power absorbed in the tissue is limited by the manufacturer according to FDA guidelines. The manufacturer has made the necessary modifications to the imaging pulse sequences to stay within the FDA guidelines and, as at 1.5T, the 3.0T has a safety mechanism to prevent an imaging sequence from starting if power absorption limits are reached.

Due to biopsy:

For the biopsy the patient will receive percutaneous epinephrine and lidocaine. Potential side effects include: pain, infection and local allergic reaction.

Anticipated adverse events are those that may be expected from tissue biopsy. This may include pain, bleeding, bruising, hemtaoma, infection or wound healing complications. In addition, if there is a complication from the biopsy (for example a hematoma), this could lead to the removal of a bigger piece of breast tissue at the time of surgery than would normally be done, which could impact the appearance of your breast after surgery.

9.0 PRIMARY OUTCOMES

We expect to determine that in a certain subset of women who have had a complete imaging response to NAC that pathology from a percutaneous magnetic resonance imaging (MRI) guided biopsy will accurately diagnose a pathologic complete response (pCR).

10.0 CRITERIA FOR REMOVAL FROM STUDY

Subjects participating may withdraw from the study at any time and for any reason.

The following events may result in the removal of patients from the study:

- Inability to comply with the protocol requirements

- Patients whose health would be jeopardized by continued participation
- Discretion of the investigator

For all participants, failure to have the planned breast surgery at MSKCC will result in removal from the study.

If a patient is removed based on the criteria above, we will replace the patient. Patients whose samples did not yield sufficient material in the judgment of the study pathologist will also be removed and replaced.

11.0 BIOSTATISTICS

Descriptive statistics will be used to summarize clinical, imaging, and pathologic parameters. The primary aim of this proposal is to estimate the negative predictive value (NPV) of a percutaneous MRI biopsy to the reference-standard breast surgery specimen. In this context NPV is defined as the number of true negatives (biopsy negative, i.e., no disease found on the percutaneous biopsy and pCR) divided by the number of all biopsy negatives. While NPV is of primary interest we will also estimate positive predictive value, sensitivity, and specificity of the biopsy.

We will enroll 25 patients with a complete imaging response. We anticipate that 14 of them will have a negative biopsy. We will consider the results promising and consider planning a confirmatory study if at least 10 of the 14 patients have a negative biopsy and complete pathological response. This decision rule will indicate further study with probability 95% if the true NPV is 85%. This probability reduces to 9% if the true NPV is 50%. We are cognizant of the fact that a decision rule of this type in a small study does not have sufficient power for some values of NPV that would be of clinical interest (such 75% or 80%), therefore we will apply this rule in the context of other dimensions of accuracy, i.e. sensitivity, specificty and positive predictive value. One example of how other dimensions will be used is the possibility that PPV is not near 100% — even if we have a high NPV a result of this sort may give us pause in taking this further. If the number of patients with negative biopsy is not 14 we will then derive rules that have the closest operating characteristics to the rule given above.

We anticipate an average accrual rate of 1 patient per month with a complete accrual of 25 patients within 24 months.

To prevent studying an imaging method that may be of no value to full accrual we will implement a futility rule halfway through the study (i.e. when 10 patients with complete imaging response are enrolled). We anticipate that seven of these patients will have negative biopsies. If one or fewer of these patients have complete pathological response than we will stop the study for futility. This decision rule will stop the study with 94% probability if the true NPV is 50% or less.

12.0 RESEARCH PARTICIPANT REGISTRATION AND RANDOMIZATION PROCEDURES

12.1 Research Participant Registration

Confirm eligibility as defined in the section entitled Criteria for Patient/Subject Eligibility.

Obtain informed consent, by following procedures defined in section entitled Informed Consent Procedures.

During the registration process registering individuals will be required to complete a protocol specific Eligibility Checklist.

All participants must be registered through the Clinical Trial Management System (CTMS) at Memorial Sloan Kettering Cancer Center. Registrations will be completed by trained research staff and must be submitted via the CTMS system (http:ctms.mskcc.org). The completed and signed informed consent form, a completed and signed Eligibility Checklist and other relevant documents must be uploaded to CTMS.

12.2 Randomization

The study will not include any randomization.

13.0 DATA MANAGEMENT ISSUES

A clinical research team will be assigned to the study. The responsibilities of the team include project compliance, data collection, abstraction and entry, data reporting (including to the institution and the sponsor), regulatory monitoring, problem resolution and prioritization and coordination of activities of the protocol study team.

The data collected for this study will be entered into a secure database at MSKCC. Data from this trial will be entered into the Clinical Research Data Base (CRDB) and a REDCap database maintained by the Principle Investigator and other members of the research study team. Source documentation will be available to support the computerized patient record.

13.1 Quality Assurance

Weekly registration reports will be generated to monitor patient accruals and completeness of registration data. Routine data quality reports will be generated to assess missing data and inconsistencies. Accrual rates and extent and accuracy of evaluations and follow-up will be monitored periodically throughout the study period.

Random-sample date quality and protocol compliance audits will be conducted by the study team, at a minimum of two times per year, more frequently if indicated.

13.2 Data and Safety Monitoring

The Data and Safety Monitoring (DSM) Plans at Memorial Sloan-Kettering Cancer Center were approved by the National Cancer Institute in September 2001. The plans address the new policies set forth by the NCI in the document entitled "Policy of the National Cancer Institute for Data and Safety Monitoring of Clinical Trials" which can be found at: *http://www.cancer.gov/clinicaltrials/conducting/dsm-guidelines/page1*. The DSM Plans at MSKCC were established and are monitored by the Office of Clinical Research. The MSKCC Data and Safety Monitoring Plans can be found on the MSKCC Intranet at: *http://smskpsps9/dept/ocr/OCR%20Website%20Documents/Clinical%20Research%20Quality%20Assurance%20(CRQA)/MSKCC%20Data%20and%20Safety%20Monitoring%20P lan.pdf*

There are several different mechanisms by which clinical trials are monitored for data, safety and quality. There are institutional processes in place for quality assurance (e.g., protocol monitoring, compliance and data verification audits, therapeutic response, and staff education on clinical research QA) and departmental procedures for quality control, plus there are two institutional committees that are responsible for monitoring the activities of our clinical trials programs. The committees: *Data and Safety Monitoring Committee (DSMC)* for Phase I and II clinical trials, and the *Data and Safety Monitoring Board (DSMB)* for Phase III clinical trials, report to the Center's Research Council and Institutional Review Board.

During the protocol development and review process, each protocol will be assessed for it's level of risk and degree of monitoring required. Every type of protocol (e.g., NIH sponsored, in-house sponsored, industrial sponsored, NCI cooperative group, etc.) will be addressed and the monitoring procedures will be established at the time of protocol activation.

14.0 PROTECTION OF HUMAN SUBJECTS

Participants will be informed that information collected during their participation in this study is considered confidential. All data gathered will be kept in a secured location and available only to members of the research study team. Findings will be presented in aggregate form only with no references made to the individual participant's data. Confidentiality of each participant's data will be protected with utmost care with all data identified solely by a code number. A list matching participant's names and code numbers will be maintained on a separate sheet of paper kept in locked storage.

Participation in and data resulting from the study will not affect patient care in any way.

Risk to Individual Study Participants: This study will involve no greater than minimal risk to the study subjects.

14.1 Privacy

MSK's Privacy Office may allow the use and disclosure of protected health information pursuant to a completed and signed Research Authorization form. The use and disclosure of protected health information will be limited to the individuals described in the Research Authorization form. A Research Authorization form must be completed by the Principal Investigator and approved by the IRB and Privacy Board (IRB/PB).

14.2 Serious Adverse Event (SAE) Reporting

An adverse event is considered serious if it results in ANY of the following outcomes:

- Death
- A life-threatening adverse event
- An adverse event that results in inpatient hospitalization or prolongation of existing hospitalization
- A persistent or significant incapacity or substantial disruption of the ability to conduct normal life functions
- A congenital anomaly/birth defect
- Important Medical Events (IME) that may not result in death, be life threatening, or require hospitalization may be considered serious when, based upon medical judgment, they may jeopardize the patient or subject and may require medical or surgical intervention to prevent one of the outcomes listed in this definition

Note: Hospital admission for a planned procedure/disease treatment is not considered an SAE.

SAE reporting is required as soon as the participant signs consent. SAE reporting is required for 30-days after the participant's last investigational treatment or intervention.

Any events that occur after the 30-day period and that are at least possibly related to protocol treatment must be reported.

If an SAE requires submission to the IRB office per IRB SOP RR-408 'Reporting of Serious Adverse Events', the SAE report must be sent to the IRB within 5 calendar days of the event. The IRB requires a Clinical Research Database (CRDB) SAE report be submitted electronically to the SAE Office as follows:

For IND/IDE trials: Reports that include a Grade 5 SAE should be sent to saegrade5@mskcc.org. All other reports should be sent to saemskind@mskcc.org.

For all other trials: Reports that include a Grade 5 SAE should be sent to saegrade5@mskcc.org. All other reports should be sent to sae@mskcc.org.

The report should contain the following information:

Fields populated from CRDB:

- Subject's initials
- Medical record number
- Disease/histology (if applicable)
- Protocol number and title

Data needing to be entered:

- The date the adverse event occurred
- The adverse event
- The grade of the event
- Relationship of the adverse event to the treatment (drug, device, or intervention)
- If the AE was expected

- The severity of the AE
- The intervention
- Detailed text that includes the following
    - A explanation of how the AE was handled
    - A description of the subject's condition
    - Indication if the subject remains on the study

- If an amendment will need to be made to the protocol and/or consent form
- If the SAE is an Unanticipated Problem

The PI's signature and the date it was signed are required on the completed report.

For IND/IDE protocols:
The CRDB SAE report should be completed as per above instructions. If appropriate, the report will be forwarded to the FDA by the SAE staff through the IND Office.

14.2.1

Any additional SAE reporting information required by the sponsor or drug supplier should be included in this section.

15.0 INFORMED CONSENT PROCEDURES

Before protocol-specified procedures are carried out, consenting professionals will explain full details of the protocol and study procedures as well as the risks involved to participants prior to their inclusion in the study. Participants will also be informed that they are free to withdraw from the study at any time. All participants must sign an IRB/PB-approved consent form indicating their consent to participate. This consent form meets the requirements of the Code of Federal Regulations and the Institutional Review Board/Privacy Board of this Center. The consent form will include the following:

1. The nature and objectives, potential risks and benefits of the intended study.
2. The length of study and the likely follow-up required.
3. Alternatives to the proposed study. (This will include available standard and investigational therapies. In addition, patients will be offered an option of supportive care for therapeutic studies.)
4. The name of the investigator(s) responsible for the protocol.
5. The right of the participant to accept or refuse study interventions/interactions and to withdraw from participation at any time.

Before any protocol-specific procedures can be carried out, the consenting professional will fully explain the aspects of patient privacy concerning research specific information. In addition to signing the IRB Informed Consent, all patients must agree to the Research Authorization component of the informed consent form.

Each participant and consenting professional will sign the consent form. The participant must receive a copy of the signed informed consent form.

16.0 REFERENCES

1. Siegel RL, Miller KD, Jemal A. Cancer statistics, 2015. CA: a cancer journal for clinicians. 2015;65(1):5-29. Epub 2015/01/07. doi: 10.3322/caac.21254. PubMed PMID: 25559415.

2. Perou CM, Sorlie T, Eisen MB, van de Rijn M, Jeffrey SS, Rees CA, Pollack JR, Ross DT, Johnsen H, Akslen LA, Fluge O, Pergamenschikov A, Williams C, Zhu SX, Lonning PE, Borresen-Dale AL, Brown PO, Botstein D. Molecular portraits of human breast tumours. Nature. 2000;406(6797):747-52. Epub 2000/08/30. doi: 10.1038/35021093. PubMed PMID: 10963602.

3. Dialani V, Chadashvili T, Slanetz PJ. Role of imaging in neoadjuvant therapy for breast cancer. Annals of surgical oncology. 2015;22(5):1416-24. Epub 2015/03/03. doi: 10.1245/s10434-015-4403-9. PubMed PMID: 25727555.

4. Mamounas EP. Impact of neoadjuvant chemotherapy on locoregional surgical treatment of breast cancer. Annals of surgical oncology. 2015;22(5):1425-33. Epub 2015/03/03. doi: 10.1245/s10434-015-4406-6. PubMed PMID: 25727558.

5. Kaufmann M, von Minckwitz G, Mamounas EP, Cameron D, Carey LA, Cristofanilli M, Denkert C, Eiermann W, Gnant M, Harris JR, Karn T, Liedtke C, Mauri D, Rouzier R, Ruckhaeberle E, Semiglazov V, Symmans WF, Tutt A, Pusztai L. Recommendations from an international consensus conference on the current status and future of neoadjuvant systemic therapy in primary breast cancer. Annals of surgical oncology. 2012;19(5):1508-16. Epub 2011/12/24. doi: 10.1245/s10434-011-2108-2. PubMed PMID: 22193884.

6. Cho N, Im SA, Park IA, Lee KH, Li M, Han W, Noh DY, Moon WK. Breast cancer: early prediction of response to neoadjuvant chemotherapy using parametric response maps for MR imaging. Radiology. 2014;272(2):385-96. Epub 2014/04/18. doi: 10.1148/radiol.14131332. PubMed PMID: 24738612.

7. Boughey JC, McCall LM, Ballman KV, Mittendorf EA, Ahrendt GM, Wilke LG, Taback B, Leitch AM, Flippo-Morton T, Hunt KK. Tumor biology correlates with rates of breast-conserving surgery and pathologic complete response after neoadjuvant chemotherapy for breast cancer: findings from the ACOSOG Z1071 (Alliance) Prospective Multicenter Clinical Trial. Annals of surgery. 2014;260(4):608-14; discussion 14-6. Epub 2014/09/10. doi: 10.1097/sla.0000000000000924. PubMed PMID: 25203877; PubMed Central PMCID: PMCPmc4159769.

8. Pinker K, Bogner W, Baltzer P, Gruber S, Bickel H, Brueck B, Trattnig S, Weber M, Dubsky P, Bago-Horvath Z, Bartsch R, Helbich TH. Improved diagnostic accuracy with multiparametric magnetic resonance imaging of the breast using dynamic contrast-enhanced magnetic resonance imaging, diffusion-weighted imaging, and 3-dimensional proton magnetic resonance spectroscopic imaging. Investigative radiology. 2014;49(6):421-30. Epub 2014/02/26. doi: 10.1097/rli.0000000000000029. PubMed PMID: 24566292.

9. Sorlie T, Perou CM, Tibshirani R, Aas T, Geisler S, Johnsen H, Hastie T, Eisen MB, van de Rijn M, Jeffrey SS, Thorsen T, Quist H, Matese JC, Brown PO, Botstein D, Lonning PE, Borresen-Dale AL. Gene expression patterns of breast carcinomas distinguish tumor subclasses with clinical implications. Proceedings of the National Academy of Sciences of the

United States of America. 2001;98(19):10869-74. Epub 2001/09/13. doi: 10.1073/pnas.191367098. PubMed PMID: 11553815; PubMed Central PMCID: PMCPmc58566.

10. Al-Hilli Z, Boughey JC, Hoskin TL, Heins CN, Hieken TJ. Increasing Use of Neoadjuvant Treatment for T1 and T2 HER2-Positive Tumors. Annals of surgical oncology. 2015;22(10):3369-75. Epub 2015/07/24. doi: 10.1245/s10434-015-4718-6. PubMed PMID: 26202564.

11. Wolff AC, Hammond ME, Hicks DG, Dowsett M, McShane LM, Allison KH, Allred DC, Bartlett JM, Bilous M, Fitzgibbons P, Hanna W, Jenkins RB, Mangu PB, Paik S, Perez EA, Press MF, Spears PA, Vance GH, Viale G, Hayes DF. Recommendations for human epidermal growth factor receptor 2 testing in breast cancer: American Society of Clinical Oncology/College of American Pathologists clinical practice guideline update. Journal of clinical oncology : official journal of the American Society of Clinical Oncology. 2013;31(31):3997-4013. Epub 2013/10/09. doi: 10.1200/jco.2013.50.9984. PubMed PMID: 24101045.

12. Puglisi F, Fontanella C, Amoroso V, Bianchi GV, Bisagni G, Falci C, Fontana A, Generali D, Gianni L, Grassadonia A, Moscetti L, Portarena I, Rossi E, Marchetti P. Current challenges in HER2-positive breast cancer. Critical reviews in oncology/hematology. 2015. Epub 2015/12/08. doi: 10.1016/j.critrevonc.2015.10.016. PubMed PMID: 26638862.

13. Rubin I, Yarden Y. The basic biology of HER2. Annals of oncology : official journal of the European Society for Medical Oncology / ESMO. 2001;12 Suppl 1:S3-8. Epub 2001/08/28. PubMed PMID: 11521719.

14. Citri A, Yarden Y. EGF-ERBB signaling: toward the systems level. Nature reviews. Molecular cell biology. 2006;7(7):505-16.Epub 2006/07/11. Doi: 10.1038/nrm1962. PubMed PMID: 16829981.

15. Deasy JO, Blanco AI, Clark VH. CERR: a computational environment for radiotherapy research. Medical physics. 2003;30(5):979-85. Epub 2003/05/30. PubMed PMID: 12773007.

16. Sutton EJ, Dashevsky BZ, Oh JH, Veeraraghavan H, Apte AP, Thakur SB, Morris EA, Deasy JO. Breast cancer molecular subtype classifier that incorporates MRI features. Journal of magnetic resonance imaging : JMRI. 2016. Epub 2016/01/13. doi: 10.1002/jmri.25119. PubMed PMID: 26756416.

17. Sutton EJ, Oh JH, Dashevsky BZ, Veeraraghavan H, Apte AP, Thakur SB, Deasy JO, Morris EA. Breast cancer subtype intertumor heterogeneity: MRI-based features predict results of a genomic assay. Journal of magnetic resonance imaging : JMRI. 2015;42(5):1398-406. Epub 2015/04/09. doi: 10.1002/jmri.24890. PubMed PMID: 25850931.

17.0 APPENDICES

None
