# Supplementary material for: Accuracy of Magnetic Resonance Imaging–Guided Biopsy to Verify Breast Cancer Pathologic Complete Response After Neoadjuvant Chemotherapy: A Nonrandomized Controlled Trial
Source: JAMA Netw Open. 2021 Jan 15;4(1):e2034045. doi: 10.1001/jamanetworkopen.2020.34045 (PMC7811182; doi:10.1001/jamanetworkopen.2020.34045)
Supplement: Supplement 2. — eFigure. ROC Curve for the Logistic Regression Model to Measure the Diagnostic Accuracy of MRI Biopsy in Detecting pCR [file jamanetwopen-e2034045-s002.pdf]

Supplementary Online Content

Sutton EJ, Braunstein LZ, El-Tamer MB, et al. Accuracy of magnetic resonance imaging–guided biopsy to verify breast cancer pathologic complete response after neoadjuvant chemotherapy: a nonrandomized controlled trial. *JAMA Netw Open*. 2021;4(1):e2034045. doi:10.1001/jamanetworkopen.2020.34045

eFigure. ROC Curve for the Logistic Regression Model to Measure the Diagnostic Accuracy of MRI Biopsy in Detecting pCR

This supplementary material has been provided by the authors to give readers additional information about their work.

© 2021 Sutton EJ et al. *JAMA Network Open.*

eFigure. ROC Curve for the Logistic Regression Model to Measure the Diagnostic Accuracy of MRI Biopsy in Detecting pCR

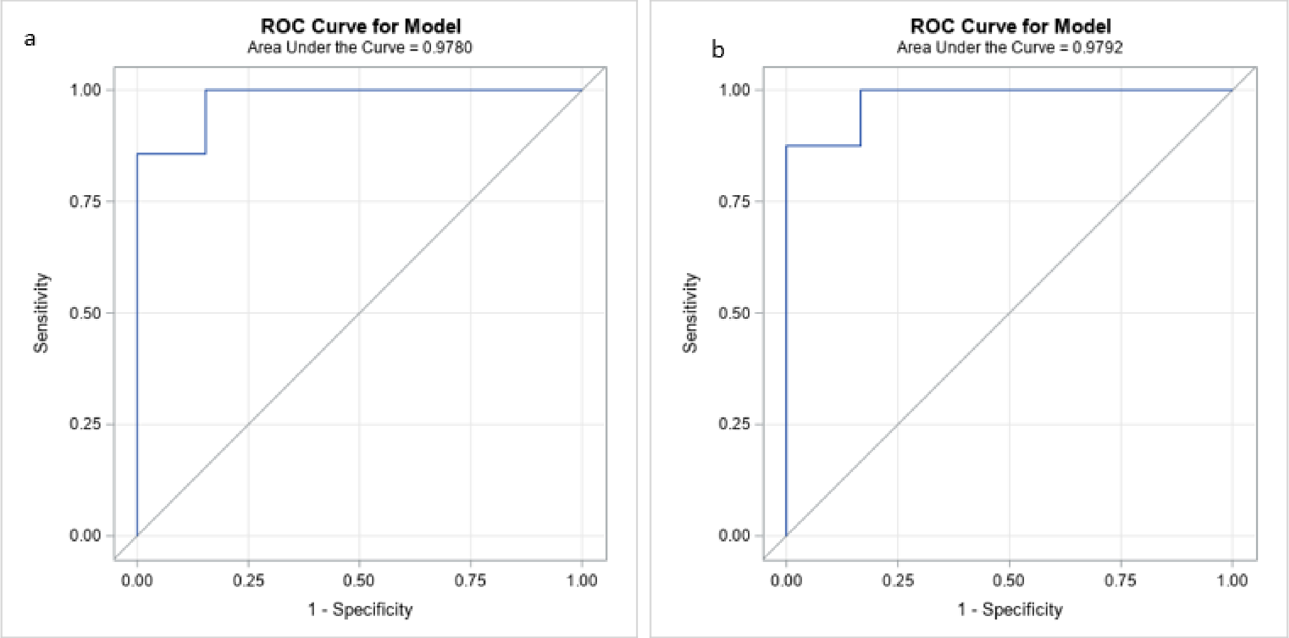

(a) ROC curve using pCR Definition I (no residual invasive cancer). (b) ROC curve using pCR Definition II (no residual invasive or in situ cancer). It can be seen that the area under the ROC curve for Definitions I and II is 0.98 (95% CI: 0.93–1.00) for both when the data is rounded to two decimal places. These results are indicative of the strong ability of MRI biopsy to differentiate patients with pCR apart from patients with no-pCR.

Abbreviations: ROC, receiver operating characteristic; pCR, pathologic complete response

© 2021 Sutton EJ et al. *JAMA Network Open.*
