# Supplementary material for: Accuracy of Magnetic Resonance Imaging–Guided Biopsy to Verify Breast Cancer Pathologic Complete Response After Neoadjuvant Chemotherapy: A Nonrandomized Controlled Trial
Source: JAMA Netw Open. 2021 Jan 15;4(1):e2034045. doi: 10.1001/jamanetworkopen.2020.34045 (PMC7811182; doi:10.1001/jamanetworkopen.2020.34045)
Supplement: Supplement 3. — Data Sharing Statement [file jamanetwopen-e2034045-s003.pdf]

Data Sharing Statement

Sutton. Accuracy of Magnetic Resonance Imaging-Guided Biopsy to Verify Breast Cancer Pathologic Complete Response After Neoadjuvant Chemotherapy. *JAMA Netw Open*. Published January 15, 2021. doi:10.1001/jamanetworkopen.2020.34045

Data
Data available: Yes
Data types: Other (please specify)
Additional Information: The protocol summary, a statistical summary, and informed consent form will be made available on clinicaltrials.gov when required as a condition of Federal awards, other agreements supporting the research and/or as otherwise required.
How to access data: Requests may be made to: suttone@mskcc.org
When available: With publication

Supporting Documents
Document types: None

Additional Information
Who can access the data: Data will be shared with qualified researchers whose proposed use of the data has been approved
Types of analyses: For any reasonable purpose
Mechanisms of data availability: Requests for deidentified individual participant data reported in the manuscript will be shared under the terms of a Data Use Agreement and may only be used for approved proposals.
